# Supplementary material for: Integrated Analysis of the Lung Microbiome and Metabolome Reveals Associations Between Amino Acid Metabolism and Pulmonary Fibrosis in a Bleomycin-Induced Mouse Model
Source: Int J Mol Sci. 2026 Jun 30;27(13):5895. doi: 10.3390/ijms27135895 (PMC13362081; doi:10.3390/ijms27135895)
Supplement: Supplementary file 1 [file ijms-27-05895-s001.zip › result/Met_results_dir.html]

Directory Tree


# Directory Tree

../results
  
 ├── 1.MetQuant-QC:代谢物定性定量结果目录
  
 ├── 2.MetAnnotation:代谢物注释结果目录
  
 │ ├── KEGG
  
 │ ├── HMDB
  
 │ ├── Lipidmaps
  
 │
  
 ├── 3.MetDiffScreening:差异代谢物筛选结果目录  
 │ ├── B\_14d.vs.C\_14d
  
 │ ├── B\_28d.vs.C\_28d
  
 │
  
 ├── 4.MetDiffAnalysis:差异代谢物分析结果目录
  
 │ ├── Box\_diff
  
 │ ├── Violin\_diff
  
 │ ├── Kmeans\_diff
  
 │ ├── Heatmap\_diff
  
 │ ├── Venn\_diff
  
 │ ├── B\_14d.vs.C\_14d
  
 │ ├── B\_28d.vs.C\_28d
  
 │
  
 ├── 5.MetKeggEnrichment:差异代谢物KEGG分析结果目录
  
 │ ├── B\_14d.vs.C\_14d
  
 │ ├── B\_28d.vs.C\_28d
  
 │
  
 ├── 6.MetGSEA:GSEA富集分析结果目录
  
 │ ├── B\_14d.vs.C\_14d
  
 │ ├── B\_28d.vs.C\_28d

---

tree v1.5.3 © 1996 - 2009 by Steve Baker and Thomas Moore   
HTML output hacked and copyleft © 1998 by Francesc Rocher   
Charsets / OS/2 support © 2001 by Kyosuke Tokoro
